# Novel genotypes and phenotypes in Snijders Blok-Campeau syndrome caused by *CHD3* mutations

**DOI:** 10.3389/fgene.2024.1347933

**Published:** 2024-07-10

**Authors:** Yuanyuan Gao, Pei Wang, Mengying Chen, Kexin Pang, Yifan Sun, Bixia Zheng, Taisong Li, Hongmei Zhang, Min Zhu

**Affiliations:** ^1^ Department of Rehabilitation, Children’s Hospital of Nanjing Medical University, Nanjing, China; ^2^ Department of Children Healthcare, Children’s Hospital of Nanjing Medical University, Nanjing, China; ^3^ Nanjing Key Laboratory of Pediatrics, Children’s Hospital of Nanjing Medical University, Nanjing, China; ^4^ Chigene (Beijing) Translational Medical Research Center Co., Ltd., Beijing, China

**Keywords:** Snijders Blok-Campeau syndrome, *CHD3* gene, whole-exome sequencing, splicing assay, gene mutation

## Abstract

**Background:**

Snijders Blok-Campeau syndrome (SNIBCPS) is a rare genetic disorder characterized by facial abnormalities, hypotonia, macrocephaly, and global developmental delay (GDD) caused by mutations in *CHD3* gene. There is limited information on SNIBCPS and few studies on its pathogenic gene *CHD3*.

**Methods:**

We utilized whole-exome sequencing, *in vitro* minigene splicing assay analysis, and construction of protein models to validate the suspected pathogenic mutation. In addition, the PubMed database was searched using the keywords “Snijders Blok-Campeau syndrome,” “*CHD3*,” or “SNIBCPS” to summarize the gene mutations and clinical phenotypic characteristics of children with SNIBCPS.

**Results:**

We identified a non-frameshift variant c.3592_c.3606delGCCAAGAGAAAGATG, a splice site variant c.1708-1G>T, and two missense variants, c. 2954G>C (p.Arg985Pro) and c.3371C>T (p.A1124V), in *CHD3* variants with SNIBCPS. Importantly, the c.3592_c.3606delGCCAAGAGAAAGATG, c.1708-1G>T, and c.3371C > T (p.A1124V) loci were not reported, and the children in this study also had phenotypic features of unibrow, transverse palmar creases, tracheal bronchus, and hypomelanosis of Ito (HI). The c.1708-1G>T classical splicing mutation leads to abnormal shearing of mRNA, forming a truncated protein that ultimately affects gene function.

**Conclusion:**

Our findings have expanded the spectrum of genetic variants and clinical features in children with SNIBCPS. Splicing analysis of *CHD3* is an important method to understand the pathogenesis of spliced cells.

## 1 Introduction

Snijders Blok-Campeau syndrome (SNIBCPS, OMIM 618205), also referred to as the intellectual developmental disorder with macrocephaly, speech delay, and dysmorphic facies, is a rare autosomal dominant inheritable disease. SNIBCPS, first reported and named by Snijders Blok in 2018, is characterized by global developmental delay (GDD), hypotonia, impaired speech and language, intellectual disability, macrocephaly, abnormal facial features (prominent bossed forehead, mid-face hypoplasia, widely spaced eyes, and low-set ears), and abnormal behaviors such as autism spectrum disorder (ASD) (Coursimault et al., 2021; Drivas et al., 2020; Mizukami et al., 2021; Snijders Blok et al., 2018). The pathogenic gene *CHD3* has been rarely reported worldwide. Chromodomain helicase DNA-binding protein 3 (CHD3), a protein is a component of the nucleosome remodeling and deacetylase (NuRD) complex (Goodman and Bonni, 2019), which is involved in chromatin remodeling by deacetylating histones. Chromatin remodeling is essential for many processes including transcription (Pierson et al., 2019). Herein, we report four cases of SNIBCPS due to *CHD3* gene mutations and review the extant literature to improve clinicians’ understanding of the disease.

## 2 Materials and methods

This study was approved by the ethics committee of the Children’s Hospital of Nanjing Medical University. Any identifiable images or data in this article were published with written informed consent from all children and their parents.

### 2.1 Genetic testing

DNA extraction from the peripheral blood of patients and their parents and family whole-exome sequencing were performed by Chigene (Beijing) Translational Medical Research Center Co. Ltd. Using IDT’s xGen Exome Research Panel v2.0, capturing the probes for liquid hybridization with gDNA library sequences was used to build a whole exon library by capturing and enriching the DNA fragments of the target region. The library covered 19,396 gene-coding regions and some non-coding regions in the human genome with a capture interval of 51 Mb. High-throughput sequencing (PE150) was performed using the MGISEQ-T7 sequencer (MGI Tech Co., Ltd). The coverage of target sequencing was not less than 99%.

Raw data were processed using fastq to remove connectors and low-quality reads. The sequence was aligned with the NCBI reference genome GRCh37/hg19 and Burrows–Wheeler Aligner software, followed by base quality scoring recalibration and SNPs and indel calling using GATK. SNPs and indels were filtered based on sequence depth and variant quality to obtain high-quality and reliable variants. The variation annotation software independently developed by Chigene (www.chigene.org) was used to annotate the detected high-quality variants in various major databases, including dbSNP, Thousand genomes, ExAC, and ESP, in addition to other frequency databases (OMIM, HGMD, and ClinVar). With protein structure prediction software such as PROVEAN, SIFT, Polyphen2-HVAR, Polyphen2-HDIV, M-Cap, Revel, MutationTaster, and shear site prediction software (MaxEntScan), the harm was analyzed. Candidate mutations were then validated with potentially harmful effects on protein structure and function by Sanger sequencing, and the pathogenicity of variants was validated according to the standards and guidelines of the American College of Medical Genetics and Genomics.

### 2.2 Protein structure simulation

The AlphaFold template (ID: AF-Q12873-F1) of *CHD3* homologous protein (NM_001005273) was employed to predict the spatial structure of the protein before and after the missense mutation. The analysis of the three-dimensional structure of the protein was performed using ChimeraX software.

### 2.3 Minigene *in vitro* splicing assay

To construct *CHD3* wild-type (NM_001005273: c.1708-1G>T) minigene plasmids, PCR was used to amplify the genomic fragment containing the target exon (Exon11) harboring the variant along with the flanking intronic sequences from BII:1 genomic DNA. The mutant minigene plasmids were then constructed by connecting pMini-CopGFP plasmid (Hitrobio, Beijing, China) and sequence information via BamHI/XhoI double digests, following the wild-type plasmid design primer. Subsequently, wild-type and mutant plasmids were transfected into 293T cells. Then, PCR was utilized to amplify the cDNA obtained by reverse transcription of RNA in the cells and analyze and sequencing its products. The primer information is summarized in Supplementary Table S1.

### 2.4 Literature search

Three keywords, “Snijders Blok–Campeau syndrome,” “*CHD3*,” and “SNIBCPS,” were used for the literature search in PubMed. Finally, eight articles were summarized and analyzed (Coursimault et al., 2021; Drivas et al., 2020; Fan,2021; LeBreton et al., 2022; Malinger et al., 2021; Mizukami et al., 2021; Snijders Blok et al., 2018; van der Spek et al., 2022).

## 3 Results

### 3.1 Clinical reports

#### 3.1.1 Family A

**FIGURE 1:**
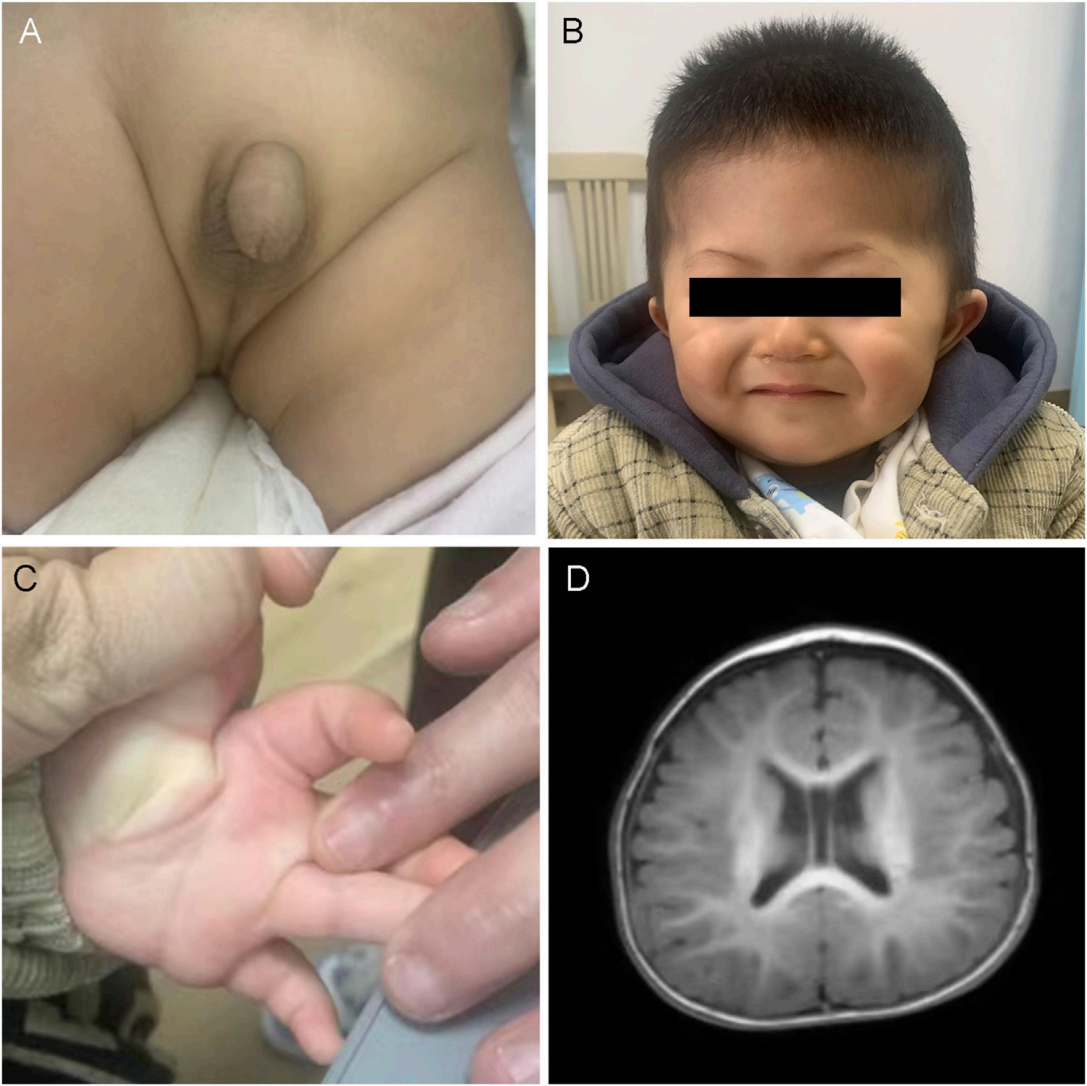
Clinical and imaging features of AII:1. **(A)** Urinary tract malformation: hypospadias. **(B)** The photo displays the facial dysmorphic features of AII:1, including a high forehead, synophrys, sparse lateral eyebrows, wide eye spacing, concave eyes, narrow lid fissures, full cheeks, mid-face recession, low nasal bridge, wide and bifid nasal tip, low ear position and thin lips. **(C)** The transverse palmar crease. **(D)** Brain MRI demonstrated that the bilateral extracerebral space was widened, the septum pellucidum was wider, and the body of the bilateral ventricles was full.

The proband, AII:1, was an 11-month-old boy who was admitted to the hospital because of unsteadiness while sitting alone. There was no family history of developmental delay; however, the daughter of the child’s aunt was suspected to have ASD. He was born to non-consanguineous parents and delivered at 34 weeks and 3 days by normal delivery, without asphyxia or convulsions at birth, and weighing 2.15 kg (<−3 standard deviations [SD]). He was admitted to our neonatal unit 1 month after birth with “bronchopneumonia” for treatment, which was diagnosed as “hypospadias, intracranial hemorrhage, left inguinal hernia, and umbilical hernia” (Figure 1A). At 6 months of age, he took four rehab therapies due to developmental delay at a local hospital. Physical examination at 11 months of age revealed a height of 74.5 cm (−1SD to 0SD), a weight of 8.65 kg (−2SD to −1SD), head circumference of 44.3 cm (−2SD to −1SD), and facial dysmorphisms (Figure 1B), including a high forehead, synophrys, sparse lateral eyebrows, ocular hypertelorism, deep-set eyes, narrow palpebral fissures, telecanthus, full cheeks, mid-face hypoplasia, broad nasal bridge, wide and bifid nasal tip, low-set ears, thin lips, and transverse palmar crease (Figure 1C). Furthermore, the patient had hypotonia. His motor skills lagged, as demonstrated by his unstable upright head posture. He was unable to crawl and required hand support while sitting alone for a while. In addition, the patient was unable to support his weight with both lower limbs when standing. He showed delayed language development: he responded when his name was called but could not understand “welcome” and “goodbye.” His right thumb was always involuntarily maintained in the adducted position. An echocardiographic examination detected no abnormalities in cardiac function. Brain magnetic resonance imaging (MRI) revealed bilateral widening of the extracerebral space in the frontotemporal roof and fullness of the body of the bilateral ventricles (Figure 1D).

#### 3.1.2 Family B

BII:1 was a girl aged 3 and 11 months and the first child of non-consanguineous Chinese parents. Notably, both her father and grandfather behaved unsocially. The mother had taken oral epilepsy medication during the early stages of pregnancy and had no significant abnormalities throughout the pregnancy. She was not premature at birth and had no hypoxic asphyxia. Physical examination at presentation revealed the presence of special features, including a high forehead, full cheeks, retracted mid-face, ocular hypertelorism, sparse lateral eyebrows, narrow palpebral fissures, low ears, wide nose bridge, and thin upper lip. Furthermore, the girl had hypotonia. Her language development was very backward, as she could only speak monosyllabic words and not short sentences. She could spontaneously pronounce words, identify her five senses, and recognize colors but could not differentiate the size and number of objects. She was inattentive, hyperactive, and constantly uncooperative in her communication, with simple instructions being completed only after repeated requests. In addition, she was diagnosed with ASD. Cranial sonography displayed no significant abnormalities.

#### 3.1.3 Family C

**FIGURE 2:**
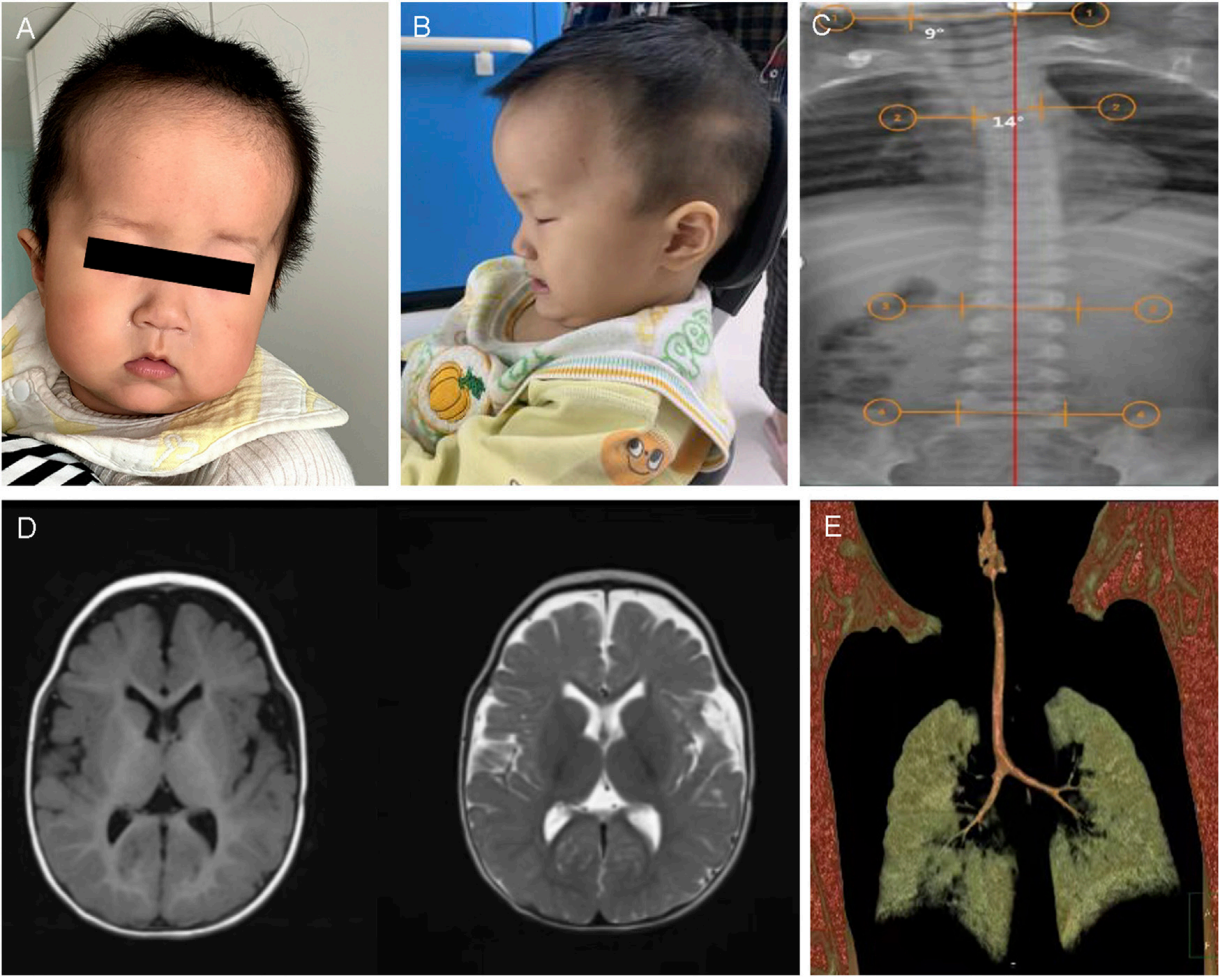
Clinical and imaging features of CII:2. **(A,B)** The picture illustrates the facial dysmorphic features consisting of a prominent bossed forehead and mid-face hypoplasia (anteroposterior and lateral film). **(C)** Anteroposterior X-ray view illustrated scoliosis of CII:2, with Cobbs angles of 10°. **(D)** Brain MRI shows a bilateral widening of the extracerebral space in the frontotemporal lobes and bilateral ventricular filling. **(E)** The chest CT scan depicted right upper pneumonia and the tracheal bronchus of the right upper lung.

CII:2 was a 10-month-old boy, the second child of non-consanguineously married Chinese parents (CII:1 is the elder brother of CII:2, who was healthy), who presented to the hospital because of motor developmental delay. The prenatal presentation of the boy was normal. The boy was born by cesarean section, with a birth length of 50 cm (−1SD to 0SD), birth weight of 3.6 kg (0SD to 1SD), and head circumference of 35 cm (0SD–1SD). Due to a history of amniotic fluid asphyxia and hypoxic–ischemic encephalopathy at birth, he was hospitalized in the neonatal unit of a local hospital after birth. He often suffered from right-sided pneumonia. When he was initially in the hospital at 10 months of age, the child was 74 cm (0SD) in height, 10 kg (0SD to 1SD) in weight, and 47 cm (1SD to 2SD) in head circumference. His dysmorphic features included a prominent forehead, full cheeks, mid-face hypoplasia, ocular hypertelorism, sparse eyebrows, low-set ears, a broad nasal bridge, and a thin upper lip (Figures 2A,B). He could only sit alone for a while and needed to bend his back to remain in the position. He sometimes responded to his name. He was rehabilitated from 10 months of age due to hypotonia. He had a stable sitting position at about 16 months and occasionally imitated articulation at 19 months. At age two, he could not walk alone or say anything meaningful. The patient was also diagnosed with ASD. An anteroposterior radiograph revealed a slight curvature of the thoracic spine (scoliosis), with a Cobb angle of 10° (Figure 2C). Brain MRI depicted an enlarged extracranial space and ventricles (Figure 2D). Chest computed tomography (CT) revealed a tracheal bronchus of the right upper lung (Figure 2E). Radiographs of the pelvis, long-term video electroencephalography, and blood genetic metabolism screening were normal. At 2 years and 3 months, he could walk and speak simple words but still had not learned to speak complete sentences.

#### 3.1.4 Family D

**FIGURE 3:**
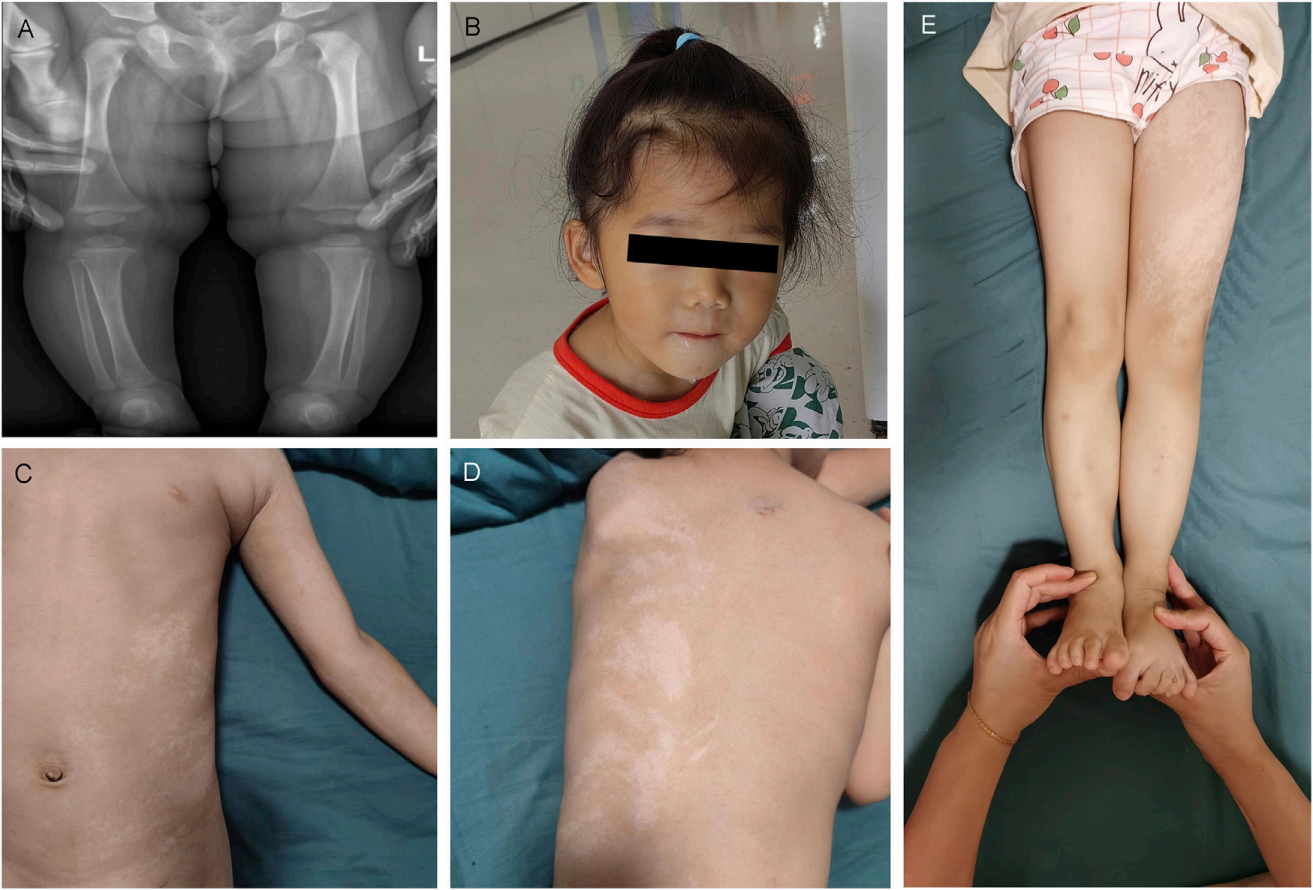
Clinical and imaging features of DII:1. **(A)** X-rays of the lower extremities of DII:1 showed unequal lengths and thicknesses of both her lower extremities without significant bone abnormalities. **(B)** DII:1 facial dysmorphic features, including high forehead, ocular hypertelorism, and low nasal bridge. **(C–E)** The loss of pigmentation within her skin, together with her longer and thicker left leg compared to her right leg.

DII:1, an 11-month-old girl, was being hospitalized for evaluation of motor and cognitive delays. She was born in a non-consanguineous family of healthy Chinese parents and had no family history of developmental delay. She suffered from aberrant fetal heartbeat and cord wrapping while fetal, had no history of turbid amniotic fluid, and was born via elective cesarean section without asphyxia hypoxia at birth. After that, an X-ray examination of the spine, pelvis, and lower limbs (Figure 3A) displayed that the length and thickness of the lower limbs were unequal, and no obvious bone abnormality was observed. At 11 months of age, she was seen in the hospital because she was still unsteadily seated alone, unable to express welcome and goodbye, and accompanied by dysmorphic facial features, including a prominent forehead, widened eye spacing, and a broad nose (Figure 3B). She exhibited GDD with hypotonia and has been undergoing regular hospital and home rehabilitation since then. She could walk alone at age two. At age three, her physical examination showed a height of 95 cm (-1SD to 0SD), a head circumference of 51.2 cm (2SD), and a weight of 13 kg (-1SD to 0SD). She was able to hold the stairs in two to three steps and scribble with a pen. However, her speech and cognition remain far behind, as evidenced by her unconscious calling for Mom and Dad. Otherwise, the patient had a slight hearing impairment. With aging, loss of pigmentation along the Blahchko line on the left side of the skin was of increasing concern. A visit to the Skin Institute confirmed the diagnosis of “hypomelanosis of Ito” (HI) (Figures 3C–E).

### 3.2 Genetic test results and pathogenicity analysis

**FIGURE 4:**
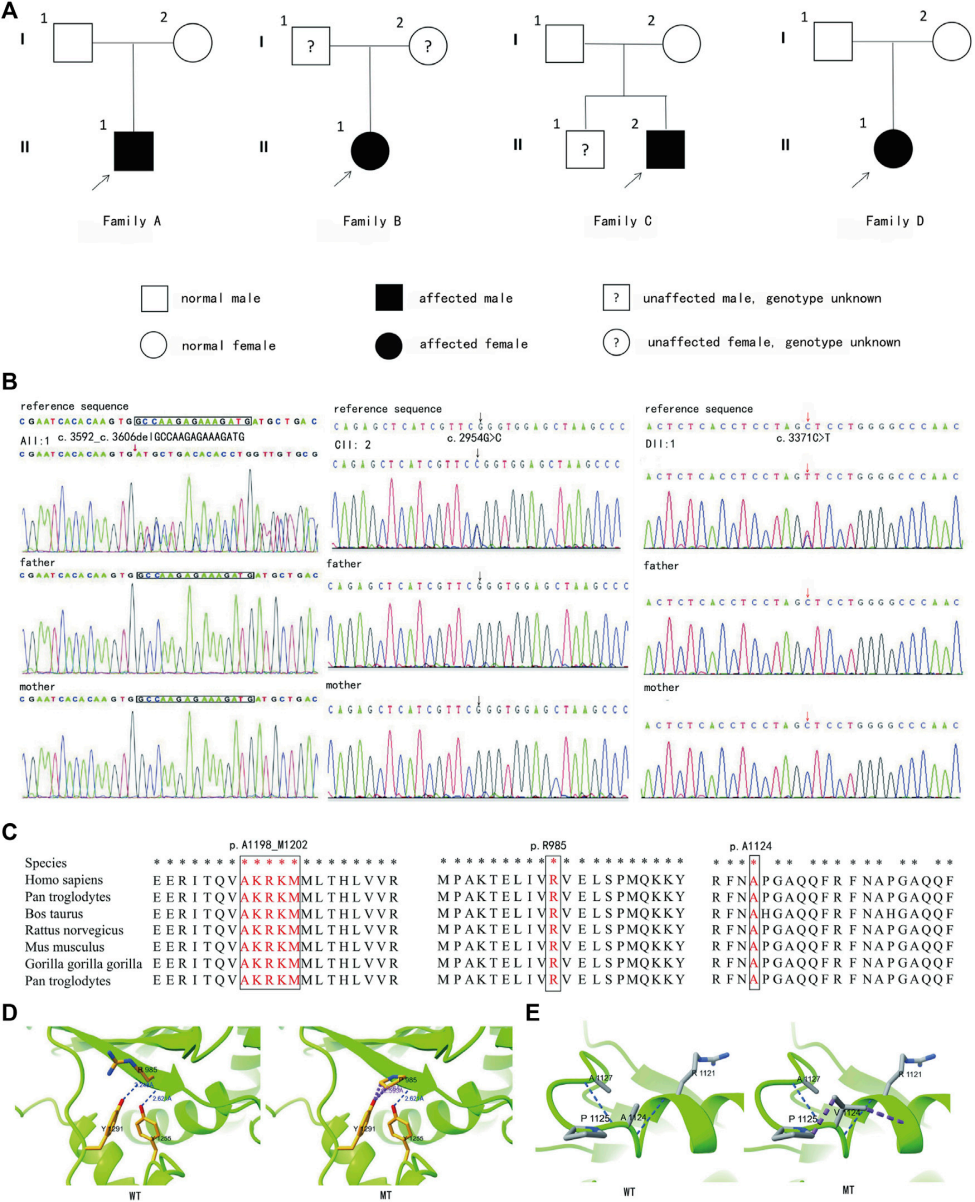
Genetic information of four families and protein model of CII:2 and DII:1. **(A)** Pedigrees of all four families. **(B)** Genetic test results of their families (Families A, C, and D). **(C)** The sites are highly conserved. **(D)** Local diagram of the number of hydrogen bonds changed before (left) and after (right) mutation in *CHD3* homologous protein (NM_001005273) p.Arg985. **(E)** Although neither hydrogen bonding nor amino acid polarity is changed before (left) and after (right) mutation, the side-chain R-groups are mutually exclusive of the other amino acid atoms (purple color), which could impact the correct folding of its active conformation and thus its function.

We sequenced the entire coding region of *CHD3* gene, including 40 exons and the sequence of the exon–intron junction region. According to the standards and guidelines of ACMG, c.1708-1G>T is a pathogenic variant, c.2954G>C (p.R985P), and c.3592_c.3606delGCCAAGAGAAAGATG (p.A1198_M1202del) are likely pathogenic variants, and c.3371C>T (p.A1124V) is a an uncertain significance variant. Amino acids are highly conserved at these loci in multiple species, as depicted in Figure 4 and Table 1.

**TABLE 1:** Genetic test results and ACMG guideline ratings.

	cDNA variants:NM_001005273.2	Amino acid variants	Type of mutation		ACMG classification	ACMG pathogenicity basis
AII:1	c.3592_c.3606delGCCAAGAGAAAGATG	p.A1198_M1202del	Nonframe shift mutation	Heterozygous *de novo* mutation	Pathogenic	PS2+PM1+PM2_Supporting + PM4
BII:1	c.1708-1G>T	-	Classical splice mutation	-	Pathogenic	PVS1+PS3+PM2_Supporting + PP3
CII: 2	c.2954G>C	p.R985P	Missense mutation	Heterozygous *de novo* mutation	Likely pathogenic	PS2+PM2_Supporting + PM5+PP3
DII:1	c.3371C>T	p.A1124V	Missense mutation	Heterozygous *de novo* mutation	Uncertain significance (VUS)	PM6+PM2_supporting + PP2+PP3

Furthermore, the three-dimensional model of the protein, observed using the ChimeraX software, indicated that p.Arg985Pro alteration resulted in the disruption of a hydrogen bond, and there was interatomic repulsion. Additionally, although p.Ala1124Val did not induce hydrogen bonding or modification in amino acid polarity, its side chain R group exhibited mutual exclusivity with other amino acid atoms. These alterations may affect protein conformation, consequently affecting its functional properties (Figure 4).

### 3.3 Minigene validation report

**FIGURE 5:**
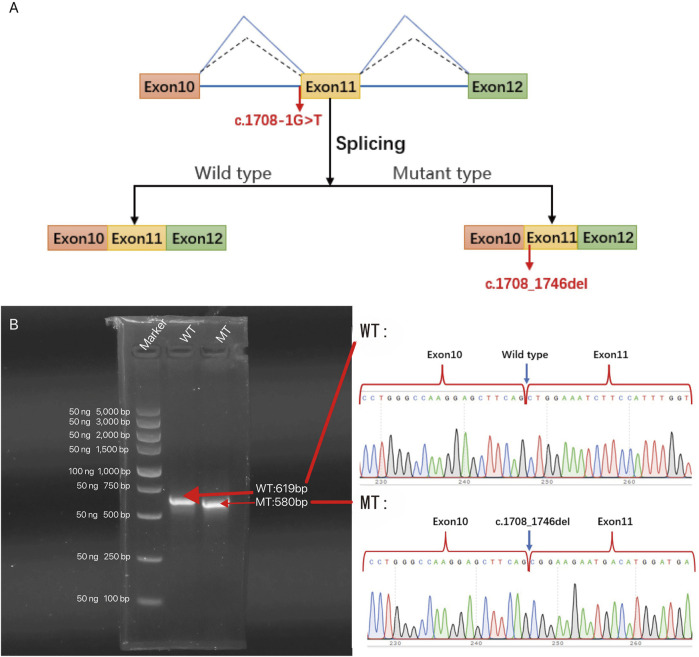
The effect of c.1708-1G>T variant on the *CHD3* gene was analyzed by minigene splicing *in vitro*: **(A)** Schematic diagram of *in vitro* splicing. **(B)** Gel electrophoresis of RT-PCR products of minigene transcription products in 293T cells (left panel). The schematic of wild type (WT) and mutant type (MT) direct sequencing (right panel). The splice site mutation c.1708-1G>T (NM_001005273.2), which causes abnormal shearing, results in a 39 bp deletion in exon 11. c. Thus, the c.1708-1G>T classical splicing mutation results in abnormal mRNA shearing, which ultimately affects gene expression.

Correct mRNA splicing is essential for eukaryotic gene expression to ensure proper function. We performed minigene validation of gene mutation in BII:1. The experimental conclusions were as follows (Figure 5): 1) The wild-type plasmid-transcribed mRNA sequences were as expected, containing the complete exons 10, 11, and 12; 2) the mutant plasmid total of one mRNA product: the mature mRNA with a 39-bp sequence partially missing in exon 11 (PCR amplification of the normal group expected sequence/target gene exon transcription sequence was 619 bp/580 bp); mRNA was represented as NM_001005273: c.1708_1746del. Protein deletion results in a truncated protein, represented as p.Leu570_Gln582del. The *CHD3* gene of BII:1 (NM_001005273.2) has a c.1708-1G>T classical splicing mutation, which leads to abnormal shearing of mRNA, resulting in the formation of truncated proteins, ultimately affecting gene function.

### 3.4 Literature review

We have summarized the clinical phenotypes (Supplementary Table S2) and genetic test results (Supplementary Figure S1) for the 111 cases reported in the literature and the four cases reported here. Among the 115 patients with SNIBCPS, we noted GDD in 93 of 110 patients (85%), language delay in 93 of 107 patients (87%), hypotonia in 70 of 96 patients (73%), and ID in 66 of 97 patients (68%). In addition, they had other common symptoms, including ocular hypertelorism in 65 of 106 patients (61%), abnormal vision in 59 of 101 patients (58%), and a prominent forehead in 59 of 104 patients (57%). Other uncommon clinical manifestations included microcephaly (three cases) and skeletal dysplasia, especially scoliosis (four cases).

All 115 patients had *CHD3* dominant heterozygous mutations. These included 70 mutations, namely, missense mutation (48), nonsense mutation (9), frameshift mutation (4), non-frameshift mutation (4), splice site mutation (2), gross mutation (2), and non-coding region mutation (1) (Supplementary Figure S1).

## 4 Discussion

SNIBCPS is a very rare neurodevelopmental disorder first reported and named by Snijders Blok in 2018. It is an autosomal dominant inheritance mainly caused by *de novo* mutations in the *CHD3* gene, which is located on the human autosome 17p13.1 and contains 40 exons. Chromodomain helicase DNA-binding proteins are adenosine triphosphate (ATP)-dependent chromatin remodeling factors. The protein encoded by the mutant gene further affects development by altering chromatin remodeling (Xie et al., 2020; Marfella and Imbalzano, 2007). CHD3 protein, a member of the second subfamily of CHD proteins, is a component of the nucleosome remodeling and deacetylase (NuRD) complex (Hoffmeister et al., 2017), which regulates gene expression in various tissues (Basta and Rauchman, 2015). NuRD complex plays a critical role in embryogenesis, is essential for normal development, and is involved in chromatin remodeling and transcriptional regulation. Developmental processes regulated by NuRD and some subunits (including CHD3) have been linked to hereditary neurodevelopmental disorders in humans. Pathogenic variants of *CHD3* are primarily associated with disruption of enzyme function, and neurodevelopmental disorders manifest as macrocephaly and intellectual disability (Pierson et al., 2019). Other features include motor and speech delay, forehead bulge, and a widened extracerebral space. The *CHD3* helicase domain (NM_001005273.1) is considered a hotspot for pathogenic variants of *CHD3* (Drivas et al., 2020). The mutation in AII:1, namely, c.3592_c.3606delGCCAAGAGAAAGATG (p.Ala1198_Met1202del), is located in the C-terminal helicase domain. However, no significant phenotypic differences were found between patients with variants in hotspots and those in other regions (Drivas et al., 2020; Snijders Blok et al., 2018), and the correlation between the genotype and phenotype of SNIBCPS remains unclear. Both activated and inactivated variants of the *CHD3* helicase domain (determined by measuring CHD3 ATPase activity and chromatin remodeling) contribute to the typical SNIBCPS phenotype. The CHD3 protein contains three alternately splicing transcripts encoding different proteins (https://www.ncbi.nlm.nih.-gov/gene/1107/). Studies have demonstrated that the transcript (NM_001005273) of *CHD3* is more highly expressed in brain tissue, possibly related to the severity of intellectual disability (Mizukami et al., 2021).AII:1 presented with GDD, speech delay, hypotonia, and typical facial features, including forehead bulge and widened eye spacing, complying with the clinical presentation of SNIBCPS (Supplementary Table S2). As in 58% of patients, cranial MRI displayed a widening of the extracerebral space, a finding observed in 58% of patients. He also suffered from urinary tract malformations, inguinal hernias, and umbilical hernias, which are uncommon clinical phenotypes in children with SNIBCPS. Notably, he also displayed synophrys and transverse palmar creases, which have not been reported previously. It is worth noting that one of his relatives had a suspected diagnosis of ASD, which served as an early warning sign. However, it is premature to determine whether the patient exhibits ASD-related symptoms at this stage, necessitating close attention and dynamic follow-up in the future. Importantly, upon discharge from the neonatal unit due to prematurity, poor weight gain, and limited response after birth coupled with macrocephaly relative to body size, doctors alerted his parents about potential developmental problems or genetic abnormalities. However, further testing did not increase until developmental delays were evident at 6 months old. The genetic abnormality was subsequently confirmed at 11 months of age.This underscores the significance of early detection and diagnosis, leading to improved prognosis.

The common clinical manifestations of BII:1 with other SNIBCPS patients include GDD, language retardation, ASD, and special facial features (Supplementary Table S2).

Patient CII:2 showed GDD, speech delay, hypotonia, ASD, peculiar facial features, and widened extracerebral gaps on cranial MRI (Supplementary Table S2), fitting SNIBCP characteristics. The presence of scoliosis in this patient may be attributed to hypotonia, and only four cases of SNIBCP children with scoliosis have been previously reported (Coursimault et al., 2021; Drivas et al., 2020; Snijders Blok et al., 2018). Additionally, a congenital malformation of the tracheal bronchus was observed on chest CT imaging in this case, which has not been previously described in association with this syndrome and increases the susceptibility to pneumonia. Former patients with SNIBCP reported clinical manifestations of the respiratory system with sleep apnea, chronic/recurrent respiratory infections, asthma, or pneumonitis (Drivas et al., 2020; Snijders Blok et al., 2018). We speculate that the tracheal bronchus may represent a novel phenotype of *CHD3* mutations affecting the respiratory system, but further research is still needed to support this hypothesis. In previous studies, the most striking finding was that eight children had different amino acid variants in p.Arg985, at the same position as this case, of which six cases were p.Arg985Trp, while the other two were p.Arg985Gln. CII:2 with the other p.Arg985 mutant had a high forehead (8/8) and mild-to-moderate intellectual disability (6/6). Consequently, we postulated that the change in p.Arg985 may not cause heavy intellectual disability (Snijders Blok et al., 2018). Nevertheless, considering the young age of CII:2, distant follow-up is necessary to track intellectual developmental levels.

The clinical manifestations of DII:1 conforming to SNIBCP include GDD, speech delay, hypotonia, hearing impairment, peculiar facial features (prominent forehead, ocular hypertelorism, and broad nose bridge), and macrocephaly (Supplementary Table S2). Her specific skin manifestations, present at birth, accrued to her unilateral trunk and extremities and were distributed asymmetrically along Blahchko’s line, otherwise normal except for differences in the amount of melanin, which supported the diagnosis of HI (a neurocutaneous syndrome). Besides, it frequently invades neurological and musculoskeletal systems, resulting in GDD, macrocephaly, inferior limb inequality, and hypotonia. Remarkably, HI is not an independent entity but rather a symptom of many different chimeric states. Although the skin lesions and other neuromusculoskeletal manifestations of DII:1 are consistent with the HI diagnosis, there is no clear explanation for hearing impairment or special facial features. As a result, we propose that the most plausible explanation for this child’s clinical presentation is SNIBCP due to mutations in the *CHD3* gene, while HI is considered a novel phenotype associated with a phenotype of SNIBCP, an observation not previously presented by others. Notably, none of the previous studies mentioned SNIBCP contributing to skin lesions, except for Theodore, who reported a few patients with skin lesions such as café patches, unusual skin folds, abnormally thick scars, and deep foot creases (Drivas et al., 2020). Nevertheless, two patients in our study exhibited different skin changes. One patient had transverse palmar creases, while the other presented with HI. In summary, we suggest these cutaneous manifestations as uncommon clinical manifestations of SNIBCP.

Currently, there is no specific treatment for SNIBCPS, and most pediatric patients experience delayed motor and cognitive development and hypotonia. Comprehensive rehabilitation therapy, including motor and cognitive training and physical therapy, can be performed to reduce the extent of developmental delay as much as possible. In our study, only CII:2 underwent systematic rehabilitation in our hospital with active out-of-hospital home-based rehabilitation. The result was a gradual improvement in his motor, cognitive, and language skills. However, it is important to acknowledge the limitations of this study, including insufficient follow-up of our patient’s post-treatment condition, which could have provided more detailed prognostic insights into disease management. Overall, maintaining long-term rehabilitation appears crucial for achieving a better prognosis despite its slow yet effective nature. Although no severe or fatal cases of SNIBCPS have been reported, early prenatal genetic screening is worth conducting to exclude *CHD3* mutations when the fetus is dysplastic or malformed. Moreover, due to the low prevalence of this disease, with only approximately 110 identified cases worldwide to date, there is potential underreporting from areas with limited medical resources. Further studies are warranted to explore the positive role of *CHD3* gene-targeting therapy in effectively managing SNIBCPS.

SNIBCPS diagnosis relies on clinical presentation and genetic testing results. There have been no reports of the mutation sites in AII:1, BII:1, and DII:1; consequently, our findings have expanded the genetic variation spectrum of SNIBCPS. Given the distinctive facial features observed in the children involved in this study and that BII:1 was markedly behind in language development, genetic testing was recommended by medical professionals. This led to identifying the mutation in *CHD3* and further diagnosis of SNIBCPS combined with clinical analysis. These findings highlight the importance of genetic screening to exclude *CHD3* mutations in children with special facial features or those that are significantly behind in their development. Additionally, AII:1, BII:1, and DII:1 infants were diagnosed with SNIBCPS, thereby requiring long-term follow-up to observe if they had missing teeth or ASD. Notably, our study achieved earlier detection and diagnosis of genetic disorders than previously reported cases (excluding an induced fetus and a 10-month-old European female infant (Malinger et al., 2021; van der Spek et al., 2022)), emphasizing the need for pediatricians to possess comprehensive knowledge and heightened awareness when identifying rare diseases in clinical practice.

In summary, we conducted whole-exome sequencing along with clinical analysis of four Chinese children with SNIBCPS, and our findings expanded both the variant spectrum and phenotypic understanding of this disease among healthcare practitioners. Furthermore, our discovery regarding the splicing analysis of *CHD3* has significant implications for understanding SNIBCPS pathogenesis.

## Data Availability

The data availability statement has been changed as follows: The data presented in the study are deposited in the Clinvar repository (https://www.ncbi.nlm.nih.gov/clinvar/), accession number SCV004031106; SCV003936085; SCV003936084; SCV003936083.
