# Supplementary material for: Novel genotypes and phenotypes in Snijders Blok-Campeau syndrome caused by CHD3 mutations
Source: Front Genet. 2024 Jul 10;15:1347933. doi: 10.3389/fgene.2024.1347933 (PMC11266126; doi:10.3389/fgene.2024.1347933)
Supplement: Supplementary file 1 [file Presentation1.zip › Supplementary Table 1.pdf]

Supplementary Table 1. Primers of Minigene In Vitro Splicing Assay.

CHD3-WT-forward primer	*CHD3*-F	AAGCTTGGTACCGAGCTCGGATCCTGCCCCGTGCTGAAGGGTCGAGTGCAGAA
CHD3-WT-reverse primer	*CHD3*-R	TTAAACGGGCCCTCTAGACTCGAGCGGTGTCTCCAGTAGCTTTGCTTATGTTC
CHD3-MT-forward primer	*CHD3*-MT-F	CCCTGCTAtCTGGAAATCTTCCATTTGGTTATG
CHD3-MT-reverse primer	*CHD3*-MT-R	ATTTCCAGaTAGCAGGGCACAGAAAAAAGGGA
identification primer	β-globin intron-F	GATATACACTGTTTGAGATGAGGA
	CHD3-CX-1R	GGCAATAAGAGAATAACAGG
sequencing primer	MiniRT-F	GGCTAACTAGAGAACCCACTGCTTA
	CHD3-RT-R	CGGTGTCTCCAGTAGCTTTGCTTATG
