# Supplementary material for: Novel genotypes and phenotypes in Snijders Blok-Campeau syndrome caused by CHD3 mutations
Source: Front Genet. 2024 Jul 10;15:1347933. doi: 10.3389/fgene.2024.1347933 (PMC11266126; doi:10.3389/fgene.2024.1347933)
Supplement: Supplementary file 1 [file Presentation1.zip › Supplementary Table 2.pdf]

Supplementary Table 2. Clinical features of 115 patients with Snijders Blok-Campeau syndrome caused by *CHD3* gene mutation.

Clinical features	Reported Cases and Ours	AII:1	BII:1	CII: 2	DII: 1
Generalized developmental delay	93/110(85%)	+	+	+	+
Language retardation	93/107(87%)	+	+	+	+
Hypotonia	70/96(73%)	+	-	+	+
Autistic Features	37/100(37%)	N/A	+	+	-
Good social contact/happy demeanor	17/3(52%)	-	-	-	-
Intellectual Disability (ID)	66/97(68%)	N/A	N/A	N/A	N/A
Severe ID	14/66(21%)	N/A	N/A	N/A	N/A
Moderate to Moderate-Severe ID	19/66(29%)	N/A	N/A	N/A	N/A
Mild ID	22/66(33%)	N/A	N/A	N/A	N/A
Seizures	16/104(15%)	-	-	-	-
Any Structural CNS abnormality	43/75(57%)	+	-	+	N/A
Widening of the extracranial space	24/44(55%)	+	-	+	N/A

Delayed myelination	5/27(19%)	-	-	-	N/A
Congenital Heart Disease (CHD)	14/101(14%)	-	N/A	-	N/A
Atrial septal defect	5/14(36%)	-	N/A	-	N/A
Ventricular septal defect	2/14(14%)	-	N/A	-	N/A
Patent ductus arteriosus	4/14(29%)	-	N/A	-	N/A
Visual abnormality	59/101(58%)	N/A	N/A	N/A	N/A
Strabismus	25/63(40%)	-	-	-	-
Cortical visual impairment	8/63(13%)	-	-	-	-
Astigmatism	7/59(12%)	N/A	N/A	N/A	N/A
Hyperopia	18/59(31%)	N/A	N/A	N/A	N/A
Myopia	10/59(17%)	N/A	N/A	N/A	N/A
Head/Face					
Macrocephaly	51/106(48%)	-	-	-	+
Microcephaly	3/106(3%)	-	-	-	-

Frontal bossing	59/104(57%)	+	+	+	+
Full Cheeks	34/69(49%)	+	-	+	-
Pointed chin	33/72(46%)	-	-	-	-
Mid-face hypoplasia	13/30(43%)	+	+	+	-
Eyes					
Ocular hypertelorism	65/106(61%)	+	+	+	+
Deep set eyes	35/70(50%)	+	-	-	-
Laterally sparse eyebrows	21/63(33%)	+	-	+	-
Narrow palpebral fissures	15/63(24%)	+	-	+	-
Telecanthus	12/29(41%)	-	-	-	-
Ears					
Post rotated ears	11/30(37%)	-	-	-	-
Low-set ears	19/64(30%)	+	+	+	-
Hearing Loss	5/69(7%)	-	-	-	+

Nose					
Broad nasal bridge	43/73(59%)	+	+	+	+
Prominent nose	10/63(16%)	-	-	-	-
Broad/Bifid nasal tip	9/60(15%)	+	-	-	-
Mouth					
Thin upper lip	43/70(61%)	+	-	+	-
Absent teeth	11/80(14%)	N/A	-	N/A	N/A
Malformations of the urinary tract or genitalia in males	12/46(26%)	+	N/A	-	-
Joit laxity	35/99(35%)	-	-	-	-

"+" = present; "-" = absent; N/A = unknown or not applicable
