# Supplementary material for: Novel genotypes and phenotypes in Snijders Blok-Campeau syndrome caused by CHD3 mutations
Source: Front Genet. 2024 Jul 10;15:1347933. doi: 10.3389/fgene.2024.1347933 (PMC11266126; doi:10.3389/fgene.2024.1347933)
Supplement: Supplementary file 1 [file Presentation1.zip › Supplementary Material.docx]

Supplementary Material

# Supplementary Figure


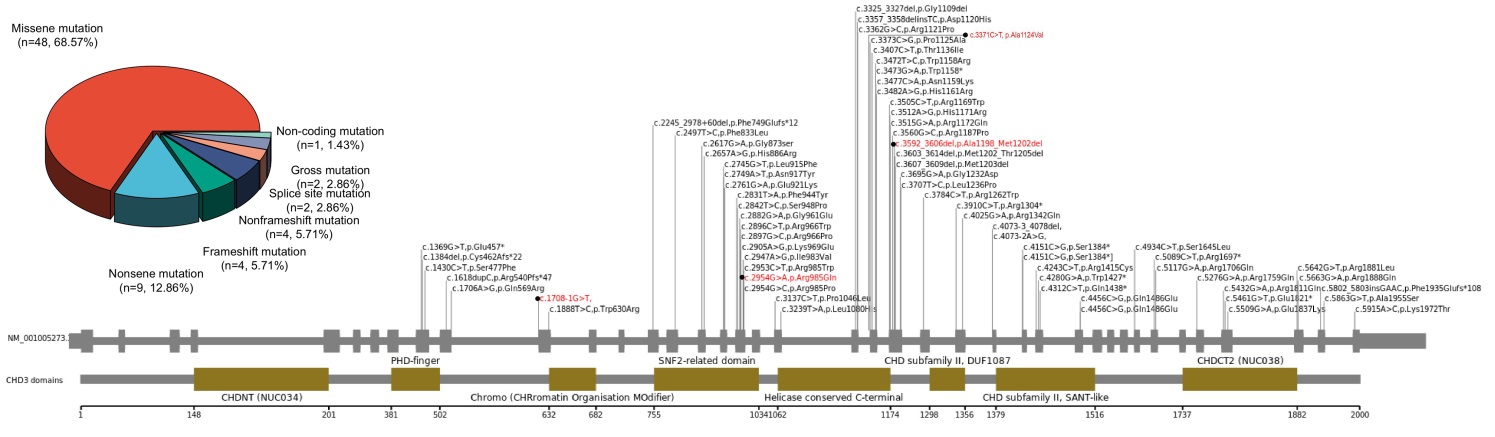


**Supplementary Figure 1.** The figure is a schematic diagram of the CHD3 structure and distribution of variation sites. The variants identified in this study are marked with solid circles.

# Supplementary Tables

## Supplementary Table 1. Primers of Minigene In Vitro Splicing Assay.

| *CHD3*-WT-forward primer | *CHD3*-F | AAGCTTGGTACCGAGCTCGGATCCTGCCCCGTGCTGAAGGGTCGAGTGCAGAA |
| --- | --- | --- |
| *CHD3*-WT-reverse primer | *CHD3*-R | TTAAACGGGCCCTCTAGACTCGAGCGGTGTCTCCAGTAGCTTTGCTTATGTTC |
| *CHD3*-MT-forward primer | *CHD3*-MT-F | CCCTGCTAtCTGGAAATCTTCCATTTGGTTATG |
| *CHD3*-MT-reverse primer | *CHD3*-MT-R | ATTTCCAGaTAGCAGGGCACAGAAAAAAGGGA |
| identification primer | β-globin intron-F | GATATACACTGTTTGAGATGAGGA |
|  | *CHD3*-CX-1R | GGCAATAAGAGAATAACAGG |
| sequencing primer | MiniRT-F | GGCTAACTAGAGAACCCACTGCTTA |
|  | *CHD3*-RT-R | CGGTGTCTCCAGTAGCTTTGCTTATG |

## Supplementary Table 2. Clinical features of 115 patients with Snijders Blok-Campeau syndrome caused by *CHD3* gene mutation.

| Clinical features | Reported Cases and Ours | AII:1 | BII:1 | CII: 2 | DII: 1 |
| --- | --- | --- | --- | --- | --- |
| Generalized developmental delay | 93/110(85%) | + | + | + | + |
| Language retardation | 93/107(87%) | + | + | + | + |
| Hypotonia | 70/96(73%) | + | - | + | + |
| Autistic Features | 37/100(37%) | N/A | + | + | - |
| Good social contact/happy demeanor | 17/3(52%) | - | - | - | - |
| Intellectual Disability (ID) | 66/97(68%) | N/A | N/A | N/A | N/A |
| Severe ID | 14/66(21%) | N/A | N/A | N/A | N/A |
| Moderate to Moderate-Severe ID | 19/66(29%) | N/A | N/A | N/A | N/A |
| Mild ID | 22/66(33%) | N/A | N/A | N/A | N/A |
| Seizures | 16/104(15%) | - | - | - | - |
| Any Structural CNS abnormality | 43/75(57%) | + | - | + | N/A |
| Widening of the extracranial space | 24/44(55%) | + | - | + | N/A |
| Delayed myelination | 5/27(19%) | - | - | - | N/A |
| Congenital Heart Disease (CHD) | 14/101(14%) | - | N/A | - | N/A |
| Atrial septal defect | 5/14(36%) | - | N/A | - | N/A |
| Ventricular septal defect | 2/14(14%) | - | N/A | - | N/A |
| Patent ductus arteriosus | 4/14(29%) | - | N/A | - | N/A |
| Visual abnormality | 59/101(58%) | N/A | N/A | N/A | N/A |
| Strabismus | 25/63(40%) | - | - | - | - |
| Cortical visual impairment | 8/63(13%) | - | - | - | - |
| Astigmatism | 7/59(12%) | N/A | N/A | N/A | N/A |
| Hyperopia | 18/59(31%) | N/A | N/A | N/A | N/A |
| Myopia | 10/59(17%) | N/A | N/A | N/A | N/A |
| Head/Face |  |  |  |  |  |
| Macrocephaly | 51/106(48%) | - | - | - | + |
| Microcephaly | 3/106(3%) | - | - | - | - |
| Frontal bossing | 59/104(57%) | + | + | + | + |
| Full Cheeks | 34/69(49%) | + | - | + | - |
| Pointed chin | 33/72(46%) | - | - | - | - |
| Mid-face hypoplasia | 13/30(43%) | + | + | + | - |
| Eyes |  |  |  |  |  |
| Ocular hypertelorism | 65/106(61%) | + | + | + | + |
| Deep set eyes | 35/70(50%) | + | - | - | - |
| Laterally sparse eyebrows | 21/63(33%) | + | - | + | - |
| Narrow palpebral fissures | 15/63(24%) | + | - | + | - |
| Telecanthus | 12/29(41%) | - | - | - | - |
| Ears |  |  |  |  |  |
| Post rotated ears | 11/30(37%) | - | - | - | - |
| Low-set ears | 19/64(30%) | + | + | + | - |
| Hearing Loss | 5/69(7%) | - | - | - | + |
| Nose |  |  |  |  |  |
| Broad nasal bridge | 43/73(59%) | + | + | + | + |
| Prominent nose | 10/63(16%) | - | - | - | - |
| Broad/Bifid nasal tip | 9/60(15%) | + | - | - | - |
| Mouth |  |  |  |  |  |
| Thin upper lip | 43/70(61%) | + | - | + | - |
| Absent teeth | 11/80(14%) | N/A | - | N/A | N/A |
| Malformations of the urinary tract or genitalia in males | 12/46(26%) | + | N/A | - | - |
| Joit laxity | 35/99(35%) | - | - | - | - |

“+” = present; “-” = absent; N/A = unknown or not applicable

# Data Availability Statement

The datasets in this study can be found in the Clinvar ( <https://www.ncbi.nlm.nih.gov/clinvar/> ), with accessions SCV004031106; SCV003936085; SCV003936084; SCV003936083.
