# Supplementary figures and images for: Novel genotypes and phenotypes in Snijders Blok-Campeau syndrome caused by CHD3 mutations
Source: Front Genet. 2024 Jul 10;15:1347933. doi: 10.3389/fgene.2024.1347933 (PMC11266126; doi:10.3389/fgene.2024.1347933)

Missense mutation  
(n=48, 68.57%)

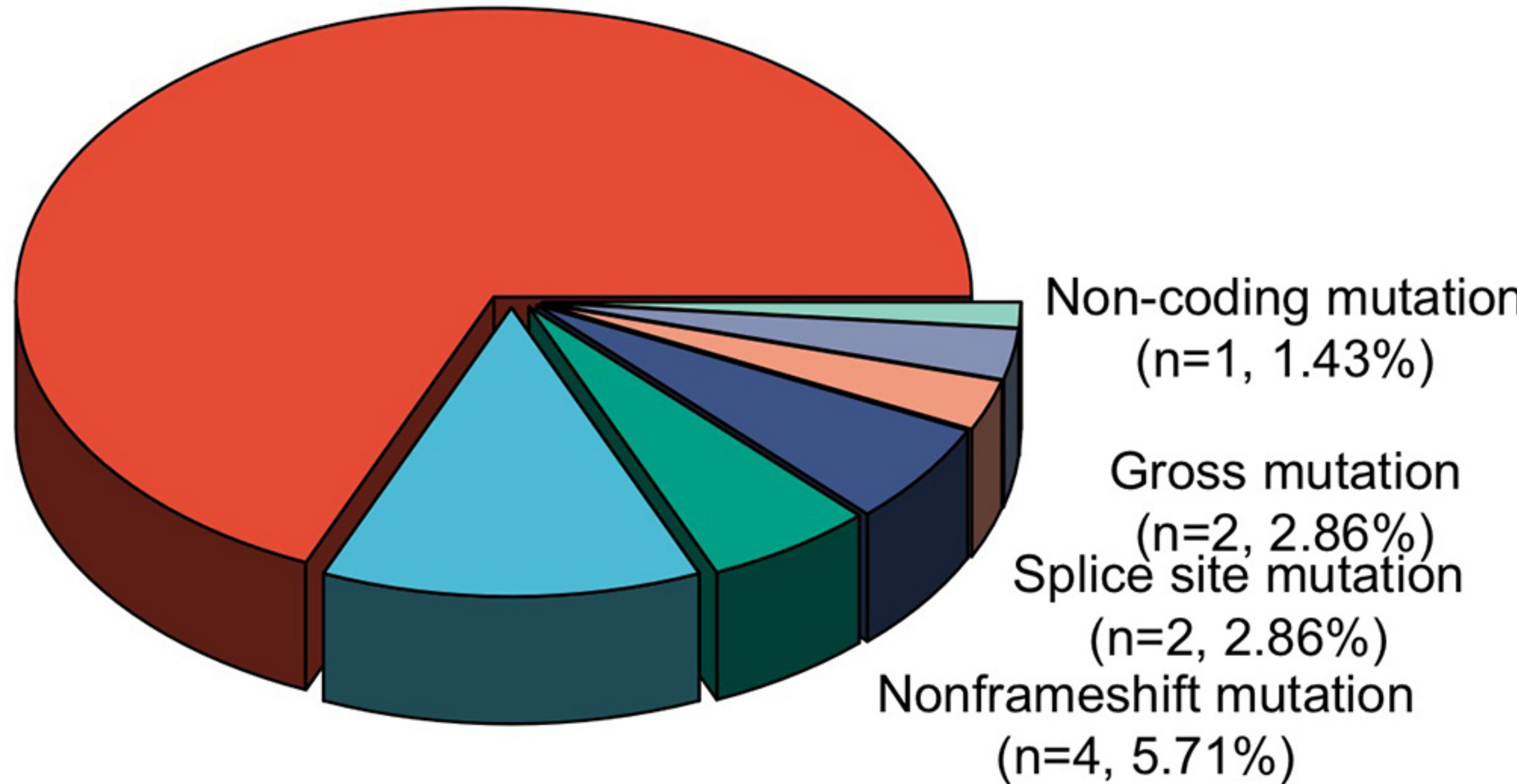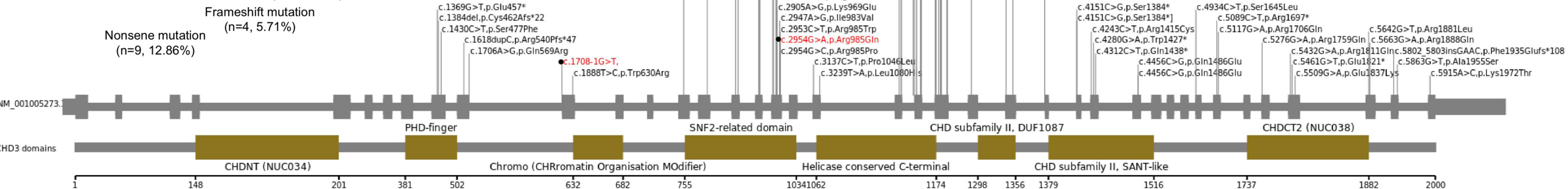

Supplement: Supplementary file 1 [file Presentation1.zip › Supplementary Figure 1.pdf]
